# Profiling the antibody response of humans protected by immunization with *Plasmodium vivax* radiation-attenuated sporozoites

**DOI:** 10.1038/s41598-024-53175-0

**Published:** 2024-02-02

**Authors:** Mary Lopez-Perez, Aarti Jain, D. Huw Davies, Juan M. Vásquez-Jiménez, Sonia M. Herrera, José Oñate, Philip L. Felgner, Sócrates Herrera, Myriam Arévalo-Herrera

**Affiliations:** 1 Malaria Vaccine and Drug Development Center (MVDC), Cali, Colombia; 2 Department Physiology & Biophysics, Vaccine R&D Center, University of California Irvine, Irvine, CA USA; 3 Imbanaco - QuironSalud, Cali, Colombia; 4 Caucaseco Scientific Research Center, Cali, Colombia

**Keywords:** Infectious diseases, Vaccines

## Abstract

Malaria sterile immunity has been reproducibly induced by immunization with *Plasmodium* radiation-attenuated sporozoites (RAS). Analyses of sera from RAS-immunized individuals allowed the identification of *P. falciparum* antigens, such as the circumsporozoite protein (CSP), the basis for the RTS, S and R21Matrix-M vaccines. Similar advances in *P. vivax* (*Pv*) vaccination have been elusive. We previously reported 42% (5/12) of sterile protection in malaria-unexposed, Duffy-positive (Fy +) volunteers immunized with *Pv*RAS followed by a controlled human malaria infection (CHMI). Using a custom protein microarray displaying 515 *Pv* antigens, we found a significantly higher reactivity to *Pv*CSP and one hypothetical protein (PVX_089630) in volunteers protected against *P. vivax* infection. In mock-vaccinated Fy + volunteers, a strong antibody response to CHMI was also observed. Although the Fy- volunteers immunized with non-irradiated *Pv*-infected mosquitoes (live sporozoites) did not develop malaria after CHMI, they recognized a high number of antigens, indicating the temporary presence of asexual parasites in peripheral blood. Together, our findings contribute to the understanding of the antibody response to *P.* *vivax* infection and allow the identification of novel parasite antigens as vaccine candidates.

**Trial registration: **ClinicalTrials.gov number: NCT 01082341.

## Introduction

Malaria continues to be an important source of morbidity and mortality globally. In 2021, about 247 million clinical cases and 619,000 malaria-related deaths were estimated worldwide, with *Plasmodium falciparum* as the most prevalent parasite species^1^. Nevertheless, 4.9 million *P. vivax* cases were estimated in the same year, mainly in the Americas, Southeast Asia, and Oceania^1^. Significant effort has been invested in *P.* *vivax* malaria research, which, despite multiple technical and financial constraints, has recently indicated the feasibility of an effective *Pv*CS-based pre-erythrocytic vaccine^2,3^. Although emulating the immune mechanisms of protection is essential for vaccine development, they remain poorly understood^4–6^.

Naturally acquired clinical immunity to malaria is a slow process that occurs after repeated exposures to the parasite in endemic areas and rapidly wanes after individuals leave the endemic sites^7^. Although sterile immunity is never achieved under natural conditions, it can be reproducibly induced by immunization via mosquito bites with radiation-attenuated sporozoites (RAS)^8–15^. This immunization approach induces immune responses that block sporozoite (spz) invasion of hepatocytes and subsequent schizogonic development in the liver. This prevents malaria disease caused by asexual parasite blood stages and further transmission mediated by sexual blood stages. Moreover, genetically (GAP) and chemically attenuated (CAP) *P. falciparum* and rodent malaria parasites have confirmed the protective efficacy of whole attenuated parasites^16–24^.

The high protective efficacy experimentally demonstrated using these whole attenuated parasites is probably due to the breadth of parasite antigens simultaneously exposed to the immune system. However, current methods to attenuate and deliver attenuated parasites remain challenging, indicating the need to further identify parasite antigens involved in protection that could be developed as subunit vaccines^21,22^. Antibodies to several *P. falciparum* antigens identified in RAS immunization as probably associated with protection have been the subject of intense research on developing subunit vaccines^21,22,25–27^. The recent approval of the *P. falciparum* RTS, S by WHO as well as the progress achieved by the *Pf*-R21/MM and, more recently, a *Pv*CS formulation underscore the great value of subunit vaccines^4,21,28–30^.

In contrast to *P. falciparum,* the overall progress in *P. vivax* vaccine development and the identification of antibodies against *P.* *vivax* has been limited to relatively few proteins made available through traditional cloning methods or peptide synthesis. Only a few of the ~ 5,500 genes encoded by the *P.* *vivax* genome have been studied as potential vaccine candidates. Currently, only three *P.* *vivax* antigens, the circumsporozoite protein (*Pv*CSP)^12,28,31–33^, sexual-stage ookinete surface protein (*Pvs*25)^2,3,34^, and *P*. *vivax* Duffy-binding protein (*Pv*DBP)^35^ have reached vaccine clinical development phases (Phase Ia, Phase IIa/b). However, several other antigens expressed on blood stages, such as AMA1, members of the MSP family, and RBP family^30,36–38^ and *Pv*CelTOS in sporozoites^30,39^ are also promising candidates currently under study. Nevertheless, which of these are responsible for protection remains unclear.

**Figure 1:**
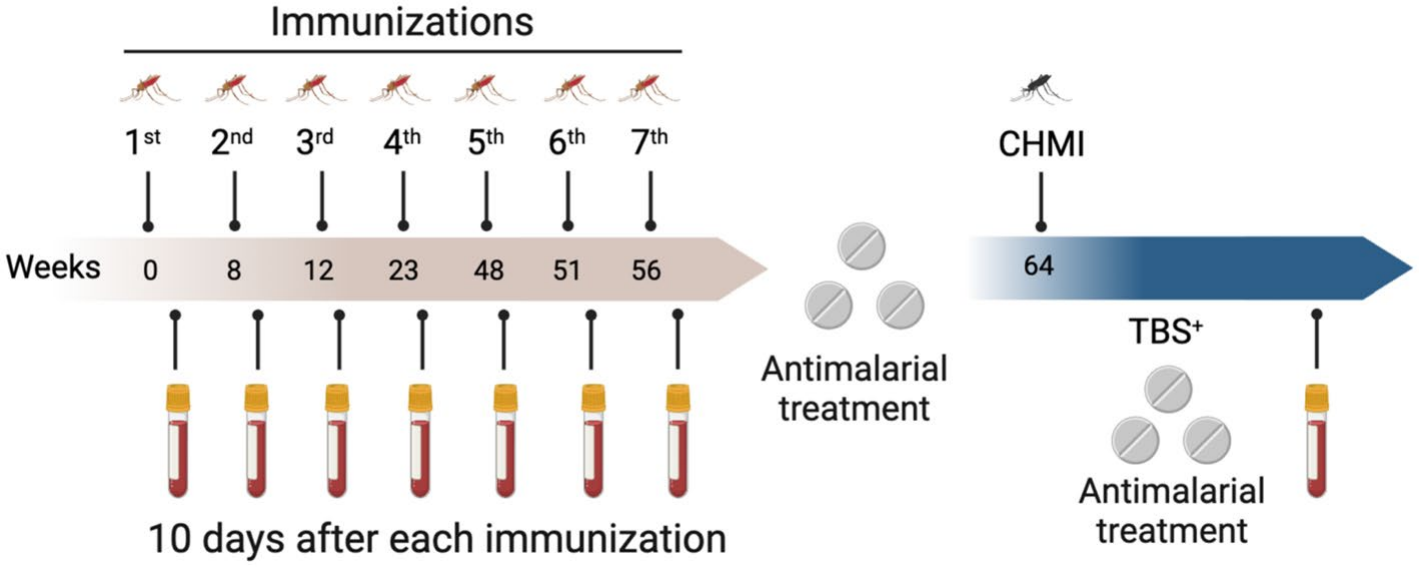
Study design and immunization schedule. Serum samples were collected from Fy + Duffy-positive individuals immunized with *Pv*RAS (n = 12) or non-infected mosquitoes (Ctl; n = 2) and Fy- Duffy negative (n = 3) ten days after each immunization. Patent blood-stage parasitemia was detected by microscopy (TBS^+^, thick blood smear) and confirmed by real-time qPCR on days 12 to 13 post-controlled malaria infection (CHMI).

To determine the feasibility of controlled vaccine clinical trials using whole attenuated *P. vivax* sporozoites, and their protective efficacy, a phase II trial was conducted^10–12^. The trial assessed the safety and protective efficacy of human immunization with *Pv*RAS in Duffy-positive (Fy +) malaria-unexposed adult volunteers, using as controls mock-vaccinated Fy + individuals and Fy- exposed to non-attenuated sporozoites (Fig. 1). This study revealed that *Pv*RAS inoculation was immunogenic, as volunteers developed antibodies and IFNγ specific to *Pv*CSP and induced sterile immunity in 42% (5/12) of the Fy + volunteers^10–12^. This trial generated valuable reagents to investigate the mechanisms of immune protection and identify relevant parasite antigens. Here we report the breadth of the serologic response using a protein microarray displaying 515 *P.* *vivax* exon products tested with serum samples collected during this trial to characterize the antibody responses induced by *Pv*RAS and their association with protection. The parasite proteins displaying the highest difference in reactivity between protected and non-protected volunteers are described and being further characterized^10^.

## Results

### Study population characteristics

Study sera from adults without previous malaria exposure, who were exposed to seven immunization doses of either *Pv*RAS, live sporozoites, or mock immunizations using non-infected mosquito bites were analyzed. Detailed information about the study participants, immunization schedule, and CHMI was previously reported^11^. Briefly, none of the volunteers developed clinical malaria or microscopic parasitemia during the immunizations; however, low levels of parasite DNA were detected by qPCR in peripheral blood after immunizations in all Fy- volunteers, which resolved spontaneously. At day 60 post-CHMI, 5/12 volunteers of the *Pv*RAS group (42%) were fully protected from the CHMI, while 2/2 mock-immunized Fy + control individuals developed parasitemia determined by microscopy and confirmed by qPCR. As expected, none of the three Fy- volunteers developed malaria infection after the CHMI.

### Antibody response after PvRAS immunization

**Figure 2:**
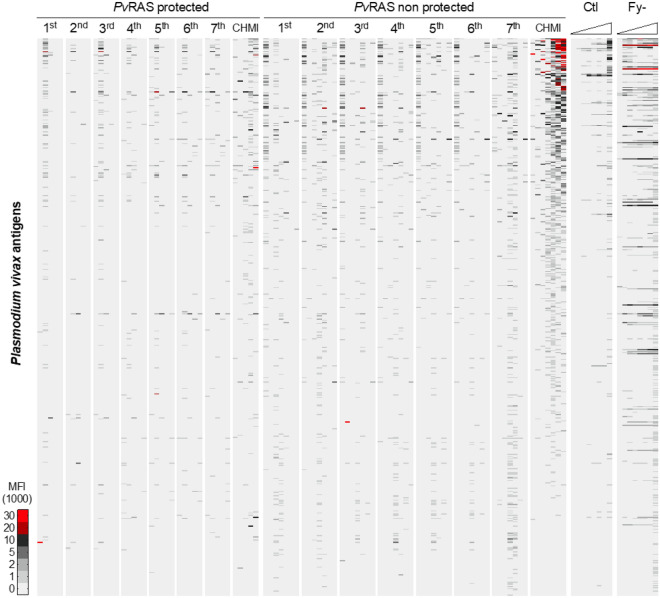
Antibody reactivity in PvRAS, control, and Fy- volunteers. Heat map showing the fluorescence intensity (MFI) ten days after each immunization and six months post-CHMI in *Pv*RAS protected (n = 5) and non-protected (n = 7). The raw signal intensity was reduced by its corresponding median IVTT-control value, and the normalized signal intensity represented by color according to the key (× 1000). All 515 antigens are shown, ranked by the average normalized intensity. The average reactivity in individuals immunized with non-infected mosquitoes (Ctl) and Duffy negative (Fy-) are shown for comparison.

**Figure 3:**
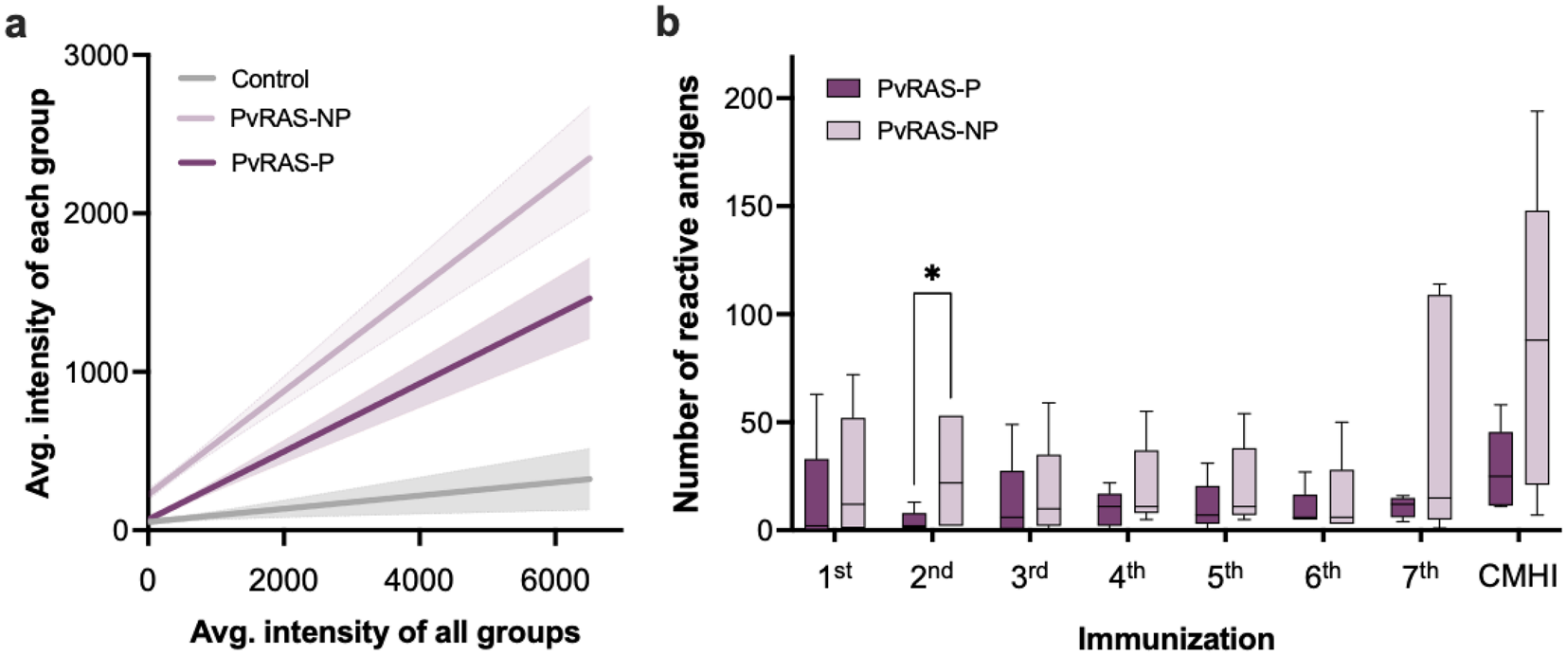
Reactivity between protected and non-protected volunteers. (**a**) Simple linear regression and 95% CI (dotted line) after the seventh immunization, in which the average reactivity to a particular antigen tested with serum samples from each group (y-axis) is plotted against the average of all groups (x-axis); the slope of the regression line is proportional to the overall breadth and intensity in each group. Data from individuals immunized with non-infected mosquitoes (Control) and *Pv*RAS protected (P) and non-protected (NP) are shown (**b**) Number of reactive antigens for protected (P) and non-protected (NP) volunteers at each immunization. Median, interquartile range (IQR), and whiskers (1.5 times the IQR) are shown. *p < 0.05 using t-test.

To identify the antibody responses induced by vaccination with *Pv*RAS, serum samples taken ten days after each of seven immunizations were probed against a custom *P.* *vivax* protein microarray^10^. Although reactivity levels were low, the fluorescence intensity increased as immunizations continued (Fig. 2). The number of reactive antigens was variable among the volunteers, ranging from 0 to 14% ten days after the first immunization and from 1.4% to 38% after the complete immunization process and CHMI (Fig. 3).

**Figure 4:**
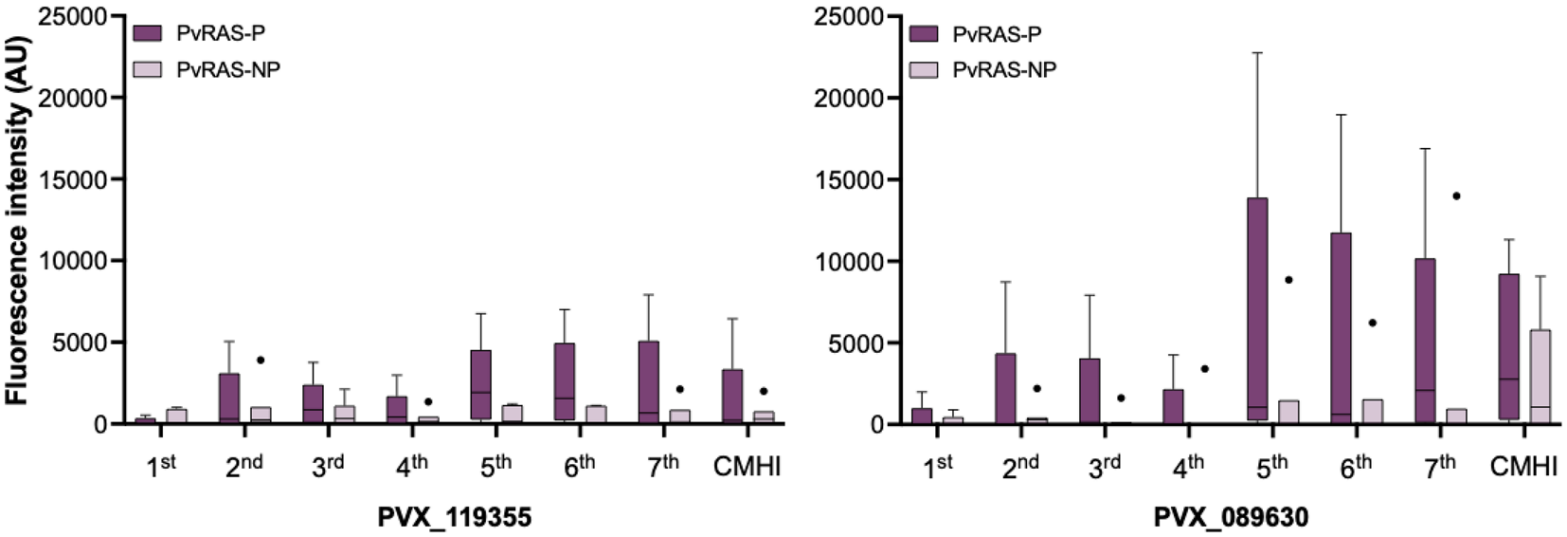
High reactivity to PvCSP in the protected PvRAS group. Reactivity to PVX_119355 (*Pv*CSP) and PVX_089630 (hypothetical protein) during the immunization schedule in protected (P) and non-protected (NP) volunteers. Median, interquartile range (IQR), and whiskers (1.5 times the IQR) are shown. Outliers are also shown.

Taking advantage of the fact that five of twelve volunteers immunized with *Pv*RAS did not develop malaria after the CHMI and aiming to identify correlates of protection, we separately analyzed the data of protected and non-protected groups. Overall, the reactivity to the antigens on the *P.* *vivax* array was lower in protected volunteers (F = 43; p < 0.0001; Fig. 3a) and they recognized fewer antigens than non-protected individuals (Fig. 3b). However, a group of ten proteins, mostly hypothetical, displayed significantly higher reactivity in the protected volunteers (Table 1, Table S1). Reactivity against *Pv*CSP (141.6 *vs.* 2170.3; p = 0.002) and one hypothetical protein (PVX_089630; 400.2 *vs*. 4523.1; p < 0.001) increased significantly at the seventh immunization but remained lower in the non-protected than in the protected volunteers during the immunization period (Table 1, Fig. 4). Likewise, we observed higher reactivity to several hypothetical proteins (n = 8) compared to *Pv*CSP in protected volunteers, encouraging further characterization, with emphasis on their potential value as *P. vivax* pre-erythrocytic vaccine candidates as reported by other studies^40,41^ (Fig. S1). When tested by ELISA using a *Pv*CSP*-*derived long synthetic peptides (*Pv*CSP-NRC), all *Pv*RAS volunteers displayed specific antibodies after the second immunization, which remained positive during the immunization period^10^; more importantly, the specific IgG1 response to these peptides was significantly higher in protected than in non-protected individuals. A group of 13 antigens with higher reactivity was identified in the non-protected volunteers six months after CHMI (Table 2).

**Table 1:** Antigens significantly recognized by the protected *Pv*RAS volunteers during the immunizations and after CHMI.

ORF PlasmoDB ID	Product description	Exon	Average normalized fluorescence intensity^a^		p value^b^
			Non-protected	Protected	
			Post-1st	Post-1st	
PVX_001000	Hypothetical protein, conserved	1 of 1	199.2	11,871.9	< 0.0001
PVX_113825	Hypothetical protein, conserved	1 of 1 S3	508.2	5051.2	< 0.0001
PVX_085025	Hypothetical protein, conserved	1 of 1 S2	303.9	2008.1	0.005

			Post-2nd	Post-2nd	
PVX_113825	Hypothetical protein, conserved	1 of 1 S3	864.4	2998.2	< 0.0001
PVX_089630	Hypothetical protein, conserved	1 of 5	477.4	1746.4	0.002
PVX_092570	Hypothetical protein, conserved	1 of 3	296.8	1341.4	0.009
PVX_094650	RAD protein (Pvfame)	2 of 2	180.7	1002.2	0.039

			Post-3rd	Post-3rd	
PVX_085025	Hypothetical protein, conserved	1 of 1 S2	156.7	4651.9	< 0.0001
PVX_113825	Hypothetical protein, conserved	1 of 1 S3	355.7	4186.5	< 0.0001
PVX_089630	Hypothetical protein, conserved	1 of 5	251.6	1652.1	0.008

			Post-4th	Post-4th	
PVX_113825	Hypothetical protein, conserved	1 of 1 S3	2477.5	3550.5	0.004
PVX_085025	Hypothetical protein, conserved	1 of 1 S2	148.7	1156.7	0.007

			Post-5th	Post-5th	
PVX_089630	Hypothetical protein, conserved	1 of 5	1487.4	5860.7	< 0.0001
PVX_091105	Membrane associated calcium-binding protein, putative	1 of 1	93.2	4111.4	< 0.0001
PVX_113825	Hypothetical protein, conserved	1 of 1 S3	675.8	3301.9	< 0.0001
PVX_119355	Circumsporozoite protein (CSP)	1 of 1	398.2	2301.1	< 0.0001

			Post-6th	Post-6th	
PVX_089630	Hypothetical protein, conserved	1 of 5	1117.0	4837.5	< 0.0001
PVX_113825	Hypothetical protein, conserved	1 of 1 S3	403.4	2582.2	< 0.0001
PVX_119355	Circumsporozoite protein (CSP)	1 of 1	375.7	2379.1	< 0.0001
PVX_084580	Kinesin, putative	3 of 3 S1	414.5	1245.4	0.016

			Post-7th	Post-7th	
PVX_089630	Hypothetical protein, conserved	1 of 5	2239.7	4523.1	< 0.0001
PVX_119355	Circumsporozoite protein (CSP)	1 of 1	538.1	2170.3	0.0007

			Post-CHMI	Post-CHMI	
PVX_110950	Hypothetical protein, conserved	1 of 1	1301.1	12,352.4	< 0.0001

^a^The raw signal intensity was reduced by its corresponding median IVTT-control value.

^b^Multiple comparison test without p-value correction.

**Table 2:** Antigens significantly recognized 6 months after the CHMI by the non-protected *Pv*RAS volunteers.

ORF PlasmoDB ID	Product description	Exon	Average normalized fluorescence intensity^a^			P value^b^
			Non-protected	Protected		
PVX_117680	Hypothetical protein	1 of 2	14,796.7	3214.1	< 0.0001	
PVX_111065	Early transcribed membrane protein (ETRAMP)	1 of 1	14,321.2	1083.8	< 0.0001	
PVX_083560	Hypothetical protein, conserved	2 of 2	13,763.9	245.1	< 0.0001	
PVX_085590	Hypothetical protein, conserved	1 of 1 S5	11,158.4	1746.7	< 0.0001	
PVX_097730	Hypothetical protein, conserved	1 of 1	11,271.9	29.7	< 0.0001	
PVX_084595	DNA replication licensing factor MCM8, putative	2 of 2 S2	11,489.1	874.3	< 0.0001	
PVX_000930	Sexual stage antigen s16, putative	1 of 1	10,366.7	192.1	< 0.0001	
PVX_099980	Merozoite surface protein 1 (MSP1)	1 of 1 S2	10,753.2	1076.9	< 0.0001	
PVX_118705	Hypothetical protein, conserved predicted Pf homolog liver stage antigen 3	1 of 1	10,001.6	410.2	< 0.0001	
PVX_090230	Early transcribed membrane protein (ETRAMP)	1 of 2	8731.8	193.1	< 0.0001	
PVX_119535	Hypothetical protein, conserved	1 of 1	8661.8	110.4	< 0.0001	
PVX_003830	Serine repeat antigen 5 (SERA5)	4 of 4	8302.4	518.4	< 0.0001	
PVX_114060	Transport protein particle (TRAPP) component, Bet3, putative		8025.4	343.9	< 0.0001	

^a^The raw signal intensity was reduced by its corresponding median IVTT-control value.

^b^Multiple comparison test without p-value correction.

In contrast, in the mock-immunized control group, there was no significant difference in the average fluorescence intensity between immunizations (Fig. 2). However, significantly increased reactivity to 18 proteins, including two members of the MSP family (*Pv*MSP1, *Pv*MSP10), SERA and eight hypothetical proteins with unknown function was observed after CHMI (Table 3).

**Table 3:** Antigens recognized by the mock-immunized (non-infected mosquitoes) Fy + control group before and after the CHMI.

ORF PlasmoDB ID	Product description	Exon	Average normalized fluorescence intensity^a^		P value^b^
			Post-7th	Post-CHMI	
PVX_118705	Hypothetical protein, conserved predicted Pf homolog liver stage antigen 3	1 of 1	0.0	28,681.7	< 0.0001
PVX_083560	Hypothetical protein, conserved	2 of 2	0.0	26,579.7	< 0.0001
PVX_001665	Hypothetical protein, conserved	1 of 1	243.9	25,159.6	< 0.0001
PVX_097730	Hypothetical protein, conserved	1 of 1	0.0	22,178.7	< 0.0001
PVX_114145	Merozoite surface protein 10 (MSP10)	1 of 1	0.0	19,252.2	< 0.0001
PVX_084595	DNA replication licensing factor MCM8, putative	2 of 2 S2	0.0	18,429.7	< 0.0001
PVX_085025	Hypothetical protein, conserved	1 of 1 S2	0.0	16,739.6	< 0.0001
PVX_121930	Hypothetical protein, conserved	2 of 2	0.0	13,723.7	< 0.0001
PVX_110880	DNA repair helicase, putative	2 of 3	0.0	11,836.7	0.0002
PVX_121885	Cytoadherence linked asexual protein, CLAG, putative	5 of 8	0.0	11,652.7	0.0002
PVX_099980	Merozoite surface protein 1 (MSP1)	1 of 1 S2	0.0	10,592.7	0.0008
PVX_084305	Hypothetical protein, conserved	1 of 1 S1	0.0	10,549.7	0.0008
PVX_084625	P-type ATPase4, putative	1 of 1 S1	0.0	8761.2	0.0053
PVX_085590	Hypothetical protein, conserved	1 of 1 S5	0.0	7942.2	0.012
PVX_091105	Membrane associated calcium binding protein, putative	1 of 1	0.0	7280.7	0.022
PVX_116915	Exported protein 2	3 of 3	0.0	6840.7	0.030
PVX_003805	Serine repeat antigen (SERA), putative	4 of 4	0.0	6552.7	0.037
PVX_084720	Hypothetical protein, conserved	1 of 1	0.0	6200.2	0.048

^a^The raw signal intensity was reduced by its corresponding median IVTT-control value.

^b^Multiple comparison test without p-value correction.

### Antibody response in Fy- individuals

**Figure 5:**
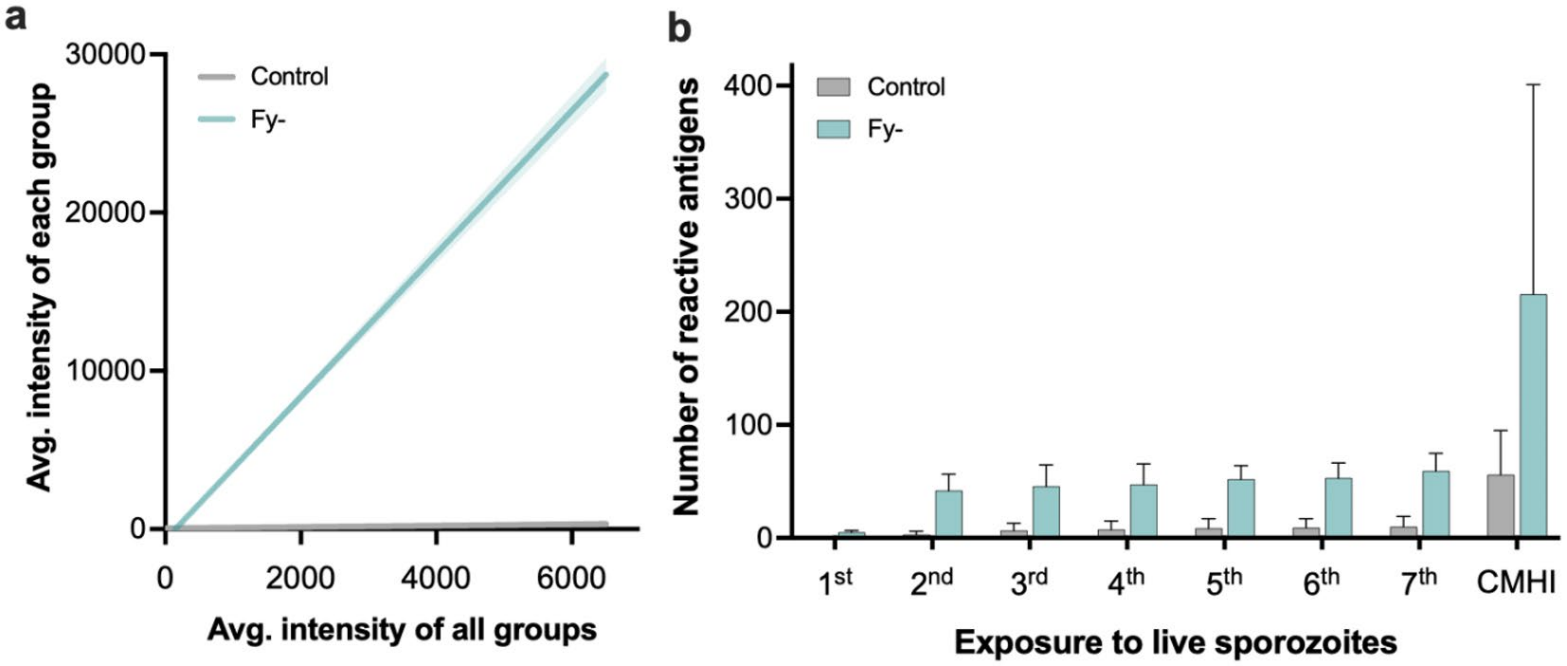
Antibody reactivity in Fy- volunteers. (**a**) Simple linear regression and 95% CI (dotted line) after the seventh round of exposure to live sporozoites, in which the average reactivity to a particular antigen tested with serum samples from each group (y-axis) is plotted against the average of all groups (x-axis); the slope of the regression line is proportional to the overall breadth and intensity in each group (F = 2829; p < 0.001). (**b**) Number of reactive antigens in volunteers exposed to non-infected mosquitoes (control) and Duffy negative (Fy-) at each round. Mean and SEM are shown.

None of the three Fy- volunteers developed patent parasitemia upon CHMI, as determined by microscopic examination of thick blood smears. However, these volunteers developed fever and malaise after the first immunization with live sporozoites by exposure to the bite of infected, non-irradiated mosquito, and parasite DNA was detected by real-time qPCR. As tested by ELISA using the *Pv*CSP-NRC peptides, seroconversion was observed in all Fy- volunteers between the second and fifth exposures. In addition, they all developed antibodies against *Pv*MSP-1 after seven exposures as tested by ELISA^10^. Moreover, although the reactivity to the antigens on the *P. vivax* array was very low after the first exposure to live sporozoites (Fig. 2), the second one significantly increased reactivity against 28 proteins (Table 4). Reactivity against antigens was maintained or increased throughout the study, peaking at the seventh exposure (Fig. 5a). The third exposure induced reactivity against two new antigens (PVX_117680 and PVX_091785); one more after the fifth one (PVX_117150); and only one (PVX_091970) after CHMI (Table S2). Although the Fy- volunteers individuals did not develop malaria after CHMI, the number of reactive antigens was higher than in the *Pv*RAS group, and as expected in the mock-immunized controls (Fig. 5b).

**Table 4:** Antigens differentially recognized by the Fy- volunteers between the first and second exposure to live sporozoites.

ORF PlasmoDB ID	Product description	Exon	Average normalized fluorescence intensity^a^		p value^b^
			Post-1st	Post-2nd	
PVX_099980	Merozoite surface protein 1 (MSP1)	1 of 1 S2	122.6	28,078.0	< 0.0001
PVX_097625	Merozoite surface protein 8 (MSP8)	1 of 1	0.0	22,691.4	< 0.0001
PVX_090090	CW type zinc finger domain containing protein	1 of 1 S1	300.3	10,973.7	< 0.0001
PVX_091970	Deoxyuridine 5'triphosphate nucleotidohydrolase, putative	1 of 1	28.4	9951.4	< 0.0001
PVX_000860	Hypothetical protein, conserved	2 of 6 S2	56.7	9722.3	< 0.0001
PVX_122810	Hypothetical protein, conserved	1 of 1 S1	177.9	8211.9	< 0.0001
PVX_003840	Serine repeat antigen 3 (SERA3)	4 of 4	125.9	7939.7	< 0.0001
PVX_000995	Transmission blocking target antigen Pfs230, putative	1 of 1	2328.3	7799.7	< 0.0001
PVX_082645	Merozoite surface protein 7 (MSP7)	1 of 1	50.1	7035.2	< 0.0001
PVX_085930	Rhoptry-associated protein 1, putative	2 of 2	1088.9	6142.4	0.0003
PVX_003770	Merozoite surface protein 5 (MSP5)	1 of 2	3.5	5928.3	< 0.0001
PVX_099520	Ubiquitin-like protein, putative	3 of 8	1136.4	5688.8	0.0009
PVX_093650	Mannose-6-phosphate isomerase, putative	1 of 1	309.5	5385.4	0.0002
PVX_119355	Circumsporozoite protein (CSP)	1 of 1	1677.7	5261.3	0.009
PVX_099315	78 kDa glucose-regulated protein precursor (GRP 78), putative	2 of 2	5.61	4693.0	0.0007
PVX_091910	kelch domain containing protein	1 of 4	285.0	4761.9	0.001
PVX_003565	Early transcribed membrane protein (ETRAMP)	1 of 1	0.0	4442.6	0.001
PVX_085960	RNA binding protein, putative		0.0	4353.6	0.001
PVX_000610	Hypothetical protein, conserved	1 of 2	0.0	4000.5	0.004
PVX_086015	Hypothetical protein, conserved	1 of 2 S6	57.3	3920.9	0.005
PVX_090215	Hypothetical membrane protein, conserved	1 of 2	96.6	3853.0	0.007
PVX_114365	Hypothetical protein, conserved	1 of 1	0.0	3832.1	0.006
PVX_118705	Hypothetical protein, predicted Pf homolog liver stage antigen 3 (LSA3)	1 of 1	169.3	3743.2	0.010
PVX_084625	P-type ATPase4, putative	1 of 1 S1	0.0	3295.4	0.017
PVX_091545	Heat shock protein 90, putative	1 of 1	0.0	3146.2	0.023
PVX_122425	M1-family amino peptidase, putative		182.3	3043.3	0.038
PVX_085590	Hypothetical protein, conserved	1 of 1 S5	0.0	2943.4	0.033
PVX_115070	Hypothetical protein, conserved	1 of 1	0.0	2855.7	0.039

^a^The raw signal intensity was reduced by its corresponding median IVTT-control value.

^b^Multiple comparison test without p-value correction.

## Discussion

The findings of this study may aid in the discovery of novel *P. vivax* antigens with potential for vaccine development with capacity to prevent the infection^34,42,43^. To our knowledge, this is the first time that sera from a *Pv*RAS clinical trial demonstrating significative sterile immunity have been screened for breadth of antibody response to identify parasite proteins associated with *P. vivax* malaria infection prevention. Although vaccines targeting other stages of the parasite cycle are important, in the case of *P. vivax,* due to the natural development of liver hypnozoites, and its consequent relapsing behavior pre-erythrocytic vaccines capable of inducing sterile immunity are of the utmost importance.

Although the original *Pv*RAS protocol aimed at delivering ten immunizations, with a total dose of ~ 100 infected mosquito bites/dose, the complex logistics imposed by the lack of *P. vivax *in vitro cultures and the need for parasites from clinical infections forced the reduction of the immunization schedule to seven doses, and the mean number of infected mosquitoes per dose from 100 to ~ 60/dose, decreasing the total dose from was 1000 to ~ 434 infective bites^11^. Because -*Pf*RAS trials are conducted using in vitro adapted parasite clones are doses and immunization timelines are readily feasible and reproducible leading to > 77–> 90% sterile protection of volunteers^44,45^. Another consequent difference of RAS vaccination between the two parasite species that while in *P. falciparum* parasites are in vitro adapted clones in *P. vivax* are wild genetically diverse parasites^12,46^. The 42% (5/12) protection attained in this *Pv*RAS vaccination trial is within the range of protection achieved with *P. falciparum*, and the apparent efficacy reduction corresponds more to the challenging conditions than a lower immune protective efficacy; unfortunately, there are no suitable means to conclusively prove this hypothesis.

Another, important difference between the RAS vaccination in the two species is the use of in vitro adapted *P. falciparum* parasites strains/clones and the need to use wild *P. vivax* isolates which are theoretically diverse parasite mixtures, despite their oligoclonal structure present in the region where parasites used here were obtained^47,48^. In addition, in this malaria endemic region the *Pv*CS-VK247 allele is highly prevalent (93%)^49^ although we did not type every parasite use for vaccination, the CHMI was conducted with *Pv*CS-VK247 which therefore, may not have influenced the protection outcome. Importantly, this study provided convenient outcome to compare the seroreactivity of protected and non-protected volunteers. In both vaccination groups, the reactivity increased as immunization progressed, confirming the dose–response effect.

This study profited from the access to Fy + and Fy − volunteers which were included to attempt dissecting the early and late immune responses elicited during the *P. vivax* liver cycle. While Fy + individuals support the complete *P. vivax* cycle, both pre-erythrocytic and erythrocytic phases, Fy- individuals allow the complete parasite liver cycle but not the erythrocytic phase which is arrested upon parasite entry in the blood circulation, due to the lack of the Duffy Antigen Receptor for Chemokines (DARC/Fy receptor) on the erythrocyte surface, required for merozoite invasion^46^; therefore, the immune response to *P. vivax* is expected to differ in these two populations. Additionally, including volunteers subjected to mock immunization allowed the dissection between parasite specific responses and the potential influence of mosquito saliva proteins in the specific immune response to the parasite antigens.

Regarding *Plasmodium* RAS vaccination, it has been demonstrated that attenuated parasites get arrested early during the liver phase and that therefore, the immune responses elicited by RAS and responsible for sterile protection presumably target parasite antigens expressed during the early hepatic schizogony. Indeed, the absence of microscopic or parasite DNA detection by qPCR during the immunization period in Fy + volunteers indirectly confirms the complete parasite radiation attenuation, and presumably the early parasite arrest^50^; in this context, no responses are expected to arise against blood parasite stages.

A relevant issue is that this study provided a convenient tool to compare the seroreactivity of protected and non-protected volunteers. In both groups, the reactivity increased as immunization progressed, confirming the dose response effect, but revealed a notably lower reactivity in the protected volunteers. However, after probing serum from both groups in the custom *P.* *vivax* protein microarray, the antibody response profile associated with sterile immunity revealed ten proteins with the highest reactivity, notably for the *Pv*CSP and the PVX_089630 proteins. Responses to these two antigens were significantly lower in the non-protected volunteers, suggesting them as potentially responsible for protection. Indeed, a *Pv*CSP formulation recently evaluated in naïve and semi-immune volunteers conferred sterile protective efficacy of 35% and 40% and overall protection of 55% and 60%, respectively^51^. The extraordinary boost in the immune response induced by the CHMI was particularly intriguing, considering that it was performed with a limited number of parasites (2–4 mosquito bites). Again, this robust antibody boosting was more evident in non-protected volunteers, which might be explained by the parasite growth and multiplication in that group, which obviously did not occur in the sterile protected volunteers.

Despite the massive inoculation of live sporozoites in Fy − control individuals, they did not develop patent microscopic parasitemia. However, parasite DNA was detected in blood by qPCR during the first three exposure doses, but not after; this observation indicated that these three rounds of exposure to live sporozoites were sufficient to induce sterile immunity^11^. In this group, seroconversion was observed between the second and fifth exposures, notably to *Pv*CSP and *Pv*MSP-1, but also to other 28 proteins. Overall, the number of reactive antigens was higher than in the *Pv*RAS group, which is reasonable as volunteers were exposed to seven doses of live sporozoites. Surprisingly, a few antigens were only recognized once during exposure or after CHMI (PVX_117680, PVX_091785, PVX_117150, PVX_091970). This group displayed an important antibody boosting upon CHMI despite the significantly lower number of sporozoites inoculated during the 2–4 mosquitoes used for the CHMI.

It has been reported that Fy- individuals from Madagascar, Cameroon, and Ethiopia may develop parasitemias when infected by *P. vivax*^52,53^. However, all Fy- volunteers in this study were refractory to blood infection by *P. vivax*. Samples from those volunteers allowed the evaluation of antibody response elicited specifically against pre-erythrocytic stages. Notably, after the second exposure, the reactivity against a group of antigens was maintained or increased through the study, suggesting that exposure to heterologous parasites induces a similar antibody response. Due to the need to use *P. vivax* parasites from natural clinical infections, each immunization is potentially a genetically different parasite, despite the limited number of circulating antigenic variants from which the blood samples to infect mosquitoes were obtained^12^. The presence of antibodies against several members of the MSP family and other antigens in the asexual blood stages, together with minor symptoms and parasite DNA found in the peripheral blood 8 to 16 days after the initial exposure to live sporozoites, indicates the presence of asexual parasites in peripheral blood. Those results agree with recent studies from two independent teams showing transient surface expression of Duffy antigen in erythroid precursors from Duffy-negative individuals and therefore supporting *P. vivax* invasion^54,55^. Since several of the antigens identified in this study are hypothetical proteins, further studies should be carried out to better characterize them.

Although counter-intuitive, observing lower reactivity in the *Pv*RAS protected group is not unusual. Recent studies on the response of individuals from malaria-endemic and non-endemic areas to *Pf*-RTS,S and *Pv*CS showed a similar hypo-responsiveness^5,51,56,57^ suggesting that individuals from malaria-endemic regions, either actively infected or not, display an altered basal immune status with a paucity of regulatory mechanisms and altered memory cell function leading to lower responsiveness to vaccines. It appears to correspond to an immunological imbalance caused by permanent exposure to malaria parasites, mosquito bites, and potentially other host and environmental factors that may influence the host’s immune response and immunity to malaria^5,6,58,59^. A similar trend was present in volunteers subjected to a *P. vivax* CHMI, in which individuals naturally exposed to *P. vivax* malaria with parasitemia and no fever (i.e., clinically protected) had lower reactivity compared to those with fever (i.e., clinically not protected)^10^. Moreover, this finding is also consistent with those from *P. falciparum* vaccination studies in humans where protected individuals did not mount a significant antibody response to CHMI, whereas non-protected individuals had elevated signals to many blood-stage antigens^34,42^ perhaps indicating a decrease in strength and variety of exposure to antigens due to earlier control and arrest of parasitic development in protected individuals. This may also hint at the importance of the cellular immune response in achieving protection, which was not assessed in this study. The observation that in the group of non-protected individuals reacted with a larger number of antigens than protected, continues to be intriguing, but does not appear to be in conflict with the fact that in the protected group some antigens induced higher IgG response intensity.

Despite most of the reactive proteins being hypothetical, a high response was observed against *Pv*CSP, a protein initially discovered by induction of the circumsporozoite precipitation reaction by sera from mice immunized with *P. berghei*-RAS. Since its discovery, *Pf*CSP has been the most extensively studied malaria antigen, leading to the only vaccine approved by the WHO for mass use^60^. Moreover, *Pv*CSP has been the subject of extensive studies^29,32,61^ and likely represents the most advanced *P. vivax* vaccine candidate, with important protective efficacy^51^. Notably, we observed higher reactivity to several hypothetical proteins compared to *Pv*CSP in protected volunteers, encouraging further characterization, with emphasis on their potential value as *P. vivax* pre-erythrocytic vaccine candidates as reported by other studies^40,41^.

Although most antibodies are short-lived, those found six months after CHMI can be used as markers of recent malaria infection. Indeed, one of those antigens (PVX_083560) was found previously in semi-immune individuals and was higher in those without fever at day 45 after CHMI^10^, thus it might be related to clinical protection. Nevertheless, whether the antigens found here remain for a longer period or whether they protect against clinical symptoms in future episodes is unknown.

Another interesting finding was the reactivity of sera from the mock-immunized control group, which was only exposed to mosquito saliva during immunization. Despite a low reactivity and no significant difference in the average fluorescence intensity between immunizations, all volunteers presented a robust reactivity with 18 parasite proteins, after volunteers’ exposure to parasite antigens upon CHMI. Whether this corresponds to a primary response to parasite antigens or a secondary (boosting) response to cross-reacting mosquito-parasite antigens remains to be determined. It is known that mosquito saliva activates innate and adaptative immune host immune responses at the biting site by activating neutrophils, monocytes, and dendritic cells and increasing both Th1/Th2 and T cell regulatory responses^58,62^. However, little is known about parasite antigens that could be expressed in the mosquito and released in the saliva^63^. Therefore, these findings open new avenues to studying the mosquito-parasite interaction.

Apart from the studies reported here on the identification of pre-erythrocytic antigens recognized by vaccinated volunteers, previous studies have focused on the analyses of parasite antigens associated with clinical protection induced by natural exposure to the parasite in endemic areas of Papua New Guinea (PNG)^40,41^. In those studies, authors selected the 20 most likely associated with protection from a total of 342 *P. vivax* antigens, from which four were merozoite surface antigens which were also recognized by sera from our *Pv*RAS trial upon challenge and by Fy- volunteers vaccinated with live sporozoites. In addition, the *Pv*CSP that was consistently recognized from early after sporozoite immunization was also reactive by clinical samples from PNG^40,41^.

In summary, we identified a group of *P.* *vivax* antigens whose antibody responses are elevated after RAS exposure and appear to contribute to sterile immunity. We also identified candidate proteins to detect previous malaria exposure due to the more durable humoral response they elicit. Taken together, these findings contribute to understanding the antibody response to *P.* *vivax* infection, particularly to the correlates of protection. Deeper analyses are required for the identification of potential surrogate markers or signatures of immune protection using systems biology^5,30,64,65^.

## Methods

### Ethics statement

The trial, from which the samples used here were obtained, received approval from the Institutional Review Boards of Centro Médico Imbanaco and the Malaria Vaccine and Drug Development Center (MVD/CECIV, No 0104 of 2009) in Cali, Colombia. All research was conducted in strict compliance with regulations and guidelines, and approved by the same Ethics Committee, and adhered to the principles outlined in the Declaration of Helsinki. Written informed consent (IC) was obtained from all volunteers, and only samples from volunteers who authorized the use of their samples in further studies were included. The clinical trial was registered at clinicaltrials.gov (registry number NCT01082341).

### Study participants and sample collection

We used serum samples stored at the MVDC/CIV cryobank collected from volunteers participating in a former Phase II clinical trial conducted to assess the protective efficacy of *Pv*RAS immunization in adults, healthy, malaria-unexposed volunteers^11^. We analyzed samples from volunteers with a complete immunization scheme followed by a CHMI, including 12/14 Fy + experimental volunteers, known to be susceptible to *P.* *vivax* infection, who were immunized with *Pv*RAS delivered by mosquito bites, and 3/7 Fy + control volunteers exposed to the bites of non-infected mosquitoes (mock-vaccination). In addition, 3/7 Fy- volunteers, known to be refractory to the erythrocyte infection by *P. vivax* asexual blood forms (merozoites) but susceptible to sporozoite liver invasion and exposed to the bite of sporozoite infected non-irradiated mosquitoes^11^. All volunteers were subjected to seven immunizations with a mean of ~ 65 mosquito bites/dose, as shown in Fig. 1, and eight weeks after the last immunization, volunteers were subjected to a *P.* *vivax* CHMI as previously described^11^. Two weeks after the last immunization, all volunteers were treated orally with a curative dose of 25 mg/kg chloroquine, divided into three doses, and 0.5 mg/kg primaquine daily for 14 days. This was done to eliminate any sub patent *P. vivax* infections that may have developed during the immunization period and to ensure a precise evaluation of infections from CHMI. The CHMI was carried out by exposing volunteers to 2–4 non-irradiated, *P.* *vivax*-infected mosquito bites until mosquitoes were blood-engorged. Starting on day five post-infection, volunteers were followed up for the development of patent infection using microscopic examination of Giemsa-stained thick-blood smears; curative antimalarial treatment as described above was provided immediately to volunteers who developed patent parasitemia. Protected volunteers were also antimalarial drug-treated when the study was completed, at day 60 post-CHMI. Serum samples collected ten days after each immunization and six months after the CHMI were analyzed.

### Protein microarray

A custom protein microarray (*Pf*/*Pv*500) displaying 515 *P. vivax* and 500 *P. falciparum* reactive exon products expressed on pre-erythrocytic and asexual parasite blood stages was purchased from Antigen Discovery Inc. (Irvine, CA)^66^. Although volunteers’ samples were hybridized to the whole array, we only present data for *P. vivax* antigens. Microarray information is publicly available on the NCBI Gene Expression Omnibus (http://www.ncbi.nlm.nih.gov/geo/) and is accessible through accession number GPL18316. Annotation of proteins presented in this study follows gene accession numbers published on PlasmoDB (www.plasmodb.org). Of 515 *P. vivax* features on the array, 444 mapped to unique *P. vivax* proteins, of which the majority (247; 56%) were classified as hypothetical or hypothetical conserved proteins. The *P. vivax* content for the array used was down-selected from the *P. vivax* 4,506-protein microarray by probing with highly reactive sera representative of different malaria-endemic populations worldwide, as detailed previously^31,56,57,59,62,67,68^. Arrays were produced as previously described^56,69^. Briefly, the proteins were produced in an *Escherichia coli*-based cell-free in vitro transcription/translation system (IVTT RTS 100 *E. coli* HY kits; 5-Prime). Each array contained multiple (n = 24) negative reaction spots lacking plasmid template expression (IVTT-control), providing a donor-specific ‘background’ signals to normalize data between individuals. Arrays also included anti-IgG and IgG spots that served as controls for the presence of primary and secondary antibodies, respectively.

For probing, serum samples were diluted 1:100 in protein array blocking buffer (Maine Manufacturing, Sanford, ME) supplemented with *E. coli* lysate (GenScript, Piscataway, NJ) to reach a final concentration of 10 mg/mL, and pre-incubated at room temperature (RT) for 30 min. In parallel, arrays were rehydrated in a blocking buffer (without lysate) for 30 min. Arrays were probed with pre-incubated serum samples overnight at 4 °C with gentle agitation and then washed at RT five times with TBS-0.05% Tween 20 (TBST), followed by incubation with biotin-conjugated goat anti-human IgG (Jackson ImmunoResearch, West Grove, PA) diluted 1:200 in blocking buffer for 1 h at RT. After incubation with secondary antibodies, arrays were washed three times in TBST, and bound IgG was visualized using streptavidin-conjugated SureLight P-3 (Columbia Biosciences, Frederick, MD) diluted 1:1000 in blocking buffer for 45 min at RT in the dark. Arrays were washed three times with TBST, and once with water. Chips were air-dried by brief centrifugation and scanned in a GenePix 4200AL laser scanner (Molecular Devices, Sunnyvale, CA). All samples in this study were probed simultaneously on the same batch of arrays.

### Data analysis

The protein microarray data was accomplished following our previously published computational methods^10,34,56,69^. Briefly, microarray spot intensities (median fluorescence intensity, MFI) were quantified using ScanArray Express software (Perkin Elmer, Waltham, MA). The antibody reactivity analysis was carried out as follows: (i) the median background signal of IVTT-control spots was calculated for each sample; (ii) the raw signal intensity was reduced by its corresponding median IVTT-control value and defined as normalized fluorescence intensity. Samples with negative values were treated as zero fluorescence intensity. Normalized data were used for statistical analyses and figures. To identify differentially reactive proteins between groups and time points, multiple comparison tests without p-value correction were used (Prism v9.5, GraphPad Software Inc., La Jolla CA). A p-value < 0.05 was considered statistically significant. Antigens were considered reactive if the fluorescence intensity of an individual (or the average for a group of individuals) was higher than a cut-off defined as the average plus two standard deviations of the reactivity to all *P. vivax* antigens in the mock-immunized control group.

### Supplementary Information

Supplementary Information.

## Data Availability

All relevant data are within the paper and its Supplementary Information files. Microarray information is publicly available on the NCBI Gene Expression Omnibus (http://www.ncbi.nlm.nih.gov/geo/) and is accessible through accession number GPL18316. The clinical trial is registered with ClinicalTrials.gov, number NCT01082341.
