# Supplementary material for: Profiling the antibody response of humans protected by immunization with Plasmodium vivax radiation-attenuated sporozoites
Source: Sci Rep. 2024 Feb 2;14:2790. doi: 10.1038/s41598-024-53175-0 (PMC10837454; doi:10.1038/s41598-024-53175-0)
Supplement: Supplementary file 1 — Supplementary Information. [file 41598_2024_53175_MOESM1_ESM.pdf]

Supplementary Information

Figure S1. Differential recognition of most reactive antigens by protected and non-protected volunteers

Six antigens were selected for the highest reactivity and the differential values between protected (red bars) and non-protected (blue bars) volunteers. Arrows indicate antigens currently being assessed for the vaccine potential [37].

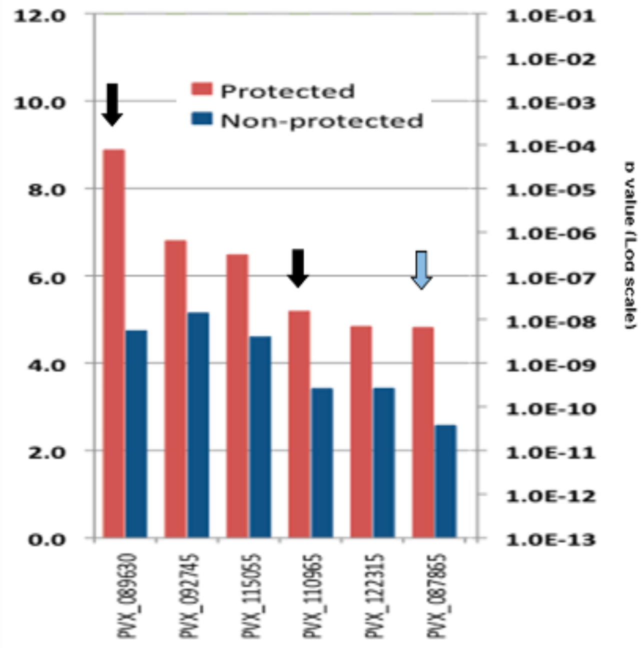

Supplementary Table 1. Antigens significantly higher by the non-protected *Pv*RAS volunteers during the immunizations.

ORF PlasmoDB ID	Average normalized fluorescence intensity [a]		p value[b]
	Non-protected	Protected	
	**Post-1st**	**Post-1st**	
PVX_099080	1973.72	102.983	0.002
PVX_100845	1746.79	88.7833	0.007
PVX_088860	1525.01	119.383	0.021
PVX_084625	1455.45	102.583	0.027
PVX_122810	1435.65	222.783	0.047
	**Post-2nd**	**Post-2nd**	
PVX_099080	3917.12	0.0	<0.0001
PVX_000610	2045.29	0.0	<0.0001
PVX_084305	1789.98	8.55833	<0.0001
PVX_084625	1723.27	0.0	<0.0001
PVX_089075	1688.94	93.1583	<0.0001
PVX_099980	1720.42	372.358	0.0007
PVX_100845	1600.13	0.0	<0.0001
PVX_096010	1535.37	152.158	0.0005
PVX_118705	1498.33	54.3083	0.0003
PVX_081830	1488.7	201.558	0.001
PVX_117150	1360.57	0.0	0.0006
PVX_092125	1380.55	66.9083	0.001
PVX_122810	1352.99	46.1583	0.001
PVX_080660	1396.69	133.158	0.002
PVX_091450	1252.45	236.242	0.011
PVX_086245	1231.69	47.7583	0.003
PVX_118040	1231.81	92.2833	0.004
PVX_116780	1159.56	0.0	0.004
PVX_088860	1100.55	66.7583	0.009
PVX_099520	1100.11	127.15	0.015
PVX_117680	1039.86	20.35	0.011
	**Post-3rd**	**Post-3rd**	
PVX_122315	5641.92	169.642	<0.0001
PVX_099080	3753.59	113.242	<0.0001
PVX_084305	1557.36	174.508	0.008
PVX_099035	1483.01	103.508	0.009
PVX_100845	1394.46	33.3083	0.009

PVX_122810	1255.15	108.308	0.029
PVX_116780	1379.81	262.508	0.033
PVX_088860	1208.93	105.908	0.035
PVX_084625	1245.22	162.708	0.039
	**Post-4th**	**Post-4th**	
PVX_080035	2203.12	16.0583	<0.0001
PVX_099980	1717.81	334.858	0.0002
PVX_118040	1711.77	122.592	<0.0001
PVX_000610	1607.05	0.0	<0.0001
PVX_099080	1597.74	110.075	<0.0001
PVX_116780	1380.99	0.0	0.0002
PVX_100845	1309.78	0.0	0.0005
PVX_099035	1276.42	140.658	0.002
PVX_088860	1235.3	125.258	0.003
PVX_122810	1210.1	0.0	0.001
PVX_084305	1202.01	100.133	0.003
PVX_085590	1182.87	0.0	0.002
	**Post-5th**	**Post-5th**	
PVX_118040	2815.75	946.067	<0.0001
PVX_000610	2673.13	0.0	<0.0001
PVX_084305	2374.08	317.242	<0.0001
PVX_122810	1694.21	0.0	<0.0001
	**Post-6th**	**Post-6th**	
PVX_118040	2936.03	1427.27	<0.0001
PVX_000610	1913.23	13.5583	<0.0001
PVX_084305	1376.74	29.875	<0.0001

[a] the raw signal intensity was reduced by its corresponding median IVTT-control value; [b] multiple comparison test without p-value correction.

Supplementary Table 2. Antigens differentially recognized by the Fy- volunteers after the third immunization.

ORF PlasmoDB ID	Product description	Exon	Average normalized fluorescence intensity [a]		p value[b]
			Post-2nd	Post-3rd	
PVX_117680	Hypothetical protein	1 of 2	3581.7	18811.0	<0.0001
PVX_091785	Translation elongation factor EF1, subunit alpha, putative	1 of 2	560.3	7538.7	0.0009
			Post-4th	Post-5th	
PVX_117150	26S proteasome subunit, putative	1 of 3	0.0	18017.8	<0.0001
			Post-7th	Post-CHMI	
PVX_091970	Deoxyuridine 5'triphosphate nucleotide hydrolase, putative	1 of 1	9407.5	16851.6	0.007

[a] the raw signal intensity was reduced by its corresponding median IVTT-control value; [b] multiple comparison test without p-value correction.
